# B7-H4 Expression Is Associated with Tumor Progression and Prognosis in Patients with Osteosarcoma

**DOI:** 10.1155/2015/156432

**Published:** 2015-04-14

**Authors:** Qiang Dong, Xinlong Ma

**Affiliations:** Department of Orthopedics, Tianjin Hospital, Tianjin Medical University, Tianjin 300211, China

## Abstract

Increasing evidences have demonstrated that B7-H4 is associated with tumor development and prognosis. However, the clinical significance of B7-H4 expression in human osteosarcoma (OS) remains unclear. The aim of present study was to examine the B7-H4 expression and to explore its contribution in OS. B7-H4 expression in OS tissues was examined by immunohistochemistry. Soluble B7-H4 (sB7-H4) levels in blood were examined by ELISA. The association of B7-H4 expression with clinicopathological factors or prognosis was statistically analyzed. Our findings demonstrated that B7-H4 expression in OS tissues was significantly higher than those in paired normal bone tissues (*P* < 0.001). sB7-H4 level in OS serum samples was significantly higher than that in healthy controls (*P* = 0.005). High B7-H4 expression in tissues and sB7-H4 level were both correlated with advanced tumor stage (*P* < 0.001, *P* = 0.017, resp.) and distant metastasis (*P* = 0.034, *P* = 0.021, resp.). Additionally, high B7-H4 expression or serum sB7-H4 levels were significantly related to poor overall survival (*P* = 0.028, *P* = 0.005, resp.). B7-H4 in tissues and serum samples were an independent factor for affecting the survival time of OS patients (*P* = 0.004, *P* = 0.041, resp.). Collectively, our data suggest that the evaluation of B7-H4 expression in tissues and blood is a useful tool for predicting the progression of osteosarcoma and prognosis.

## 1. Introduction

Osteosarcoma (OS) is the most common primary malignant bone tumor with high incidence in children and adolescents, accounting for 20–35% of all malignant primary bone tumors [1]. Despite dramatic advances in surgical techniques and chemotherapeutic treatment, the five-year survival rate for patients suffering from OS is about 50% [2, 3]. Therefore, it is necessary to improve current therapeutic modalities and to explore new biological molecular markers for predicting the progression of OS and helping targeted therapy. Recent researches have provided evidences that dysregulation of immune system may be greatly involved in the pathogenesis of OS [2].

B7-H4, also known as B7x or B7S1, is a member of the B7 family which was expressed on activated antigen presenting cells (APC) [4]. It was identified in 2003 by searching the NCBI database for sequences containing B7 extracellular Ig domains, followed by screening a placenta cDNA library [5]. B7-H4 acts through an unidentified CD28 family receptor on activated T cells and activation of B7-H4 pathway inhibits the T cell-mediated immune response [6]. Previous studies have showed that B7-H4 can regulate T cell-mediated immune response through inhibiting T cell proliferation, cytokine secretion, and the development of cytotoxicity. Recently studies have reported that B7-H4 is highly expressed in various human tumors, such as breast [7, 8], ovarian [9, 10], lung [11, 12], pancreatic [13], gastric [14, 15], and urothelial cell carcinoma [16]. In addition, the status of B7-H4 expression in tumor cells of these malignancies is closely associated with progression and prognosis [17]. Recently, soluble B7-H4 (sB7-H4) has been detected in blood samples from various cancer patients, including ovarian [18, 19], gastric [20], and renal cell carcinomas [21], and high level of sB7-H4 was a significant prognostic indicator [20].

Despite these studies, the expression pattern of B7-H4 protein and its clinical outcome in OS have not yet been investigated. Therefore, in current study, we investigated the expression levels of B7-H4 in OS surgical specimens by immunohistochemistry and circulating sB7-H4 levels in blood specimens by ELISA. Additionally, we investigated the correlation between B7-H4 expression and clinicopathological parameters and evaluated the prognostic values of B7-H4 using log-rank survival analysis.

## 2. Materials and Methods

### 2.1. Patients, Specimens, and Follow-Up

This study was approved by the Research Ethics Committee of Tianjin Hospital, China. Written informed consent was obtained from all of the patients according to the committee's regulations. All specimens were handled and made anonymous according to the ethical and legal standards. 104 paraffin-embedded OS samples and paired normal bone tissues from 2006 to 2010 were provided by the Department of Orthopedics, Tianjin Hospital, with complete histopathology and follow-up information. None of the patients received preoperative chemotherapy or radiotherapy before surgery. Clinicopathological characteristics of OS patients were detailed in Table 2. Blood specimens were collected from 86 OS patients and 50 healthy controls at the Tianjin Hospital between 2010 and 2013. Patients who had undergone any form of preoperative chemotherapy and/or radiation therapy were excluded. The healthy controls were recruited from people who came for general health examinations. Blood samples were kept at room temperature for 30 minutes, and serum was obtained after centrifugation at 4000 rpm at 4°C for 10 minutes. The serum was immediately removed and frozen on dry ice at −80°C until use. All the control subjects were matched with patient population in terms of age and sex. Selected characteristics of the cases and controls are presented in Table S1 in Supplementary Material available online at http://dx.doi.org/10.1155/2015/156432.

### 2.2. Immunohistochemistry

The paraffin-embedded specimens were cut into 5 *μ*m thick sections and then mounted on glass slides. Immunohistochemistry streptavidin peroxidase conjugated method was used to detect the expression of B7-H4. Briefly, tissues were treated with 3% H_2_O_2_ methanol at room temperature for 10 minutes and then incubated in 5% goat antiserum for 15 minutes at 37°C. After rinsing with PBS, mouse anti-human B7-H4 monoclonal antibody (clone MIH43, 1 : 200; Abcam, Cambridge, MA, USA) was added to tissues and incubated overnight at 4°C. After washing in PBS, biotin-labeled goat anti-mouse IgG was added to the sections and incubated at 37°C for 10 minutes. SP complex was added and the sections were visualized by incubating with DAB-H_2_O_2_ for 5–10 min; desired color reaction was observed when monitored with the microscope. All of the slides were counterstained with hematoxylin. Negative controls were performed by replacing the specific primary antibody with PBS.

The intensity of positive staining was measured using a computerized image system (Leica Microsystems Imaging Solutions, United Kingdom). Five fields were randomly selected, and three slides for each specimen were counted. The staining extent was scored from 0 to 3 based on the percentage of positive cells (0, <5%; 1, 5%–25%; 2, 25%–50%; 3, >50%). The intensity of staining was classified as follows: 0 point, no staining; 1 point, weak staining (light yellow); 2 points, moderate staining (brown); and 3 points, strong staining (yellowish brown), respectively. The final score of B7-H4 expression was calculated using the percent of positive cell score × staining intensity, ranging within 0–9. High B7-H4 expression level was defined as a total score ≥4, and low B7-H4 expression level was defined as a total score <4. As for the negative control, the primary antibody was replaced with PBS. When there were discrepancies between the two pathologists, the average score was used.

### 2.3. Detection of Circulating sB7-H4 by ELISA

The levels of circulating sB7-H4 in the serum were measured by enzyme-linked immunosorbent assay (ELISA) as previously described [18, 20]. Briefly, 25 *μ*L of the undiluted serum sample was added to high-binding polystyrene plates coated with capture mAb (Clone H74, eBioscience, San Diego, United States). Immobilized antigen was detected with diluted biotinylated secondary mAb (eBioscience, San Diego, United States) followed by horseradish peroxidase conjugated streptavidin (Biolegend Inc., Californian, United States). For calibration, the standards of recombinant protein and two controls were conducted in parallel with the test samples on each plate. Based on the mean value of sB7-H4 levels, we used 92.28 ng/mL as the cutoff value to divide all patients into groups with high (*n* = 36) and low (*n* = 50) sB7-H4 levels.

### 2.4. Statistical Analysis

Statistical analysis was performed with SPSS 17.0 for Windows (SPSS, Chicago, IL).

Data were expressed as means ± standard deviation (SD). The *χ* ^2^ test was used to analyze the relationship between B7-H4 expression and clinicopathological characteristics. Quantitative values were analyzed using Student's* t*-test. Survival curves were plotted using the Kaplan-Meier product-limit method, and differences between survival curves were tested using the log-rank test. The Cox regression analysis in a forward stepwise method was used to evaluate the effect of multiple independent prognostic factors on survival outcome. Differences were considered to be statistical significant when *P* value was less than 0.05.

## 3. Results

### 3.1. B7-H4 Expression in Human OS Tissues

**Figure 1:**
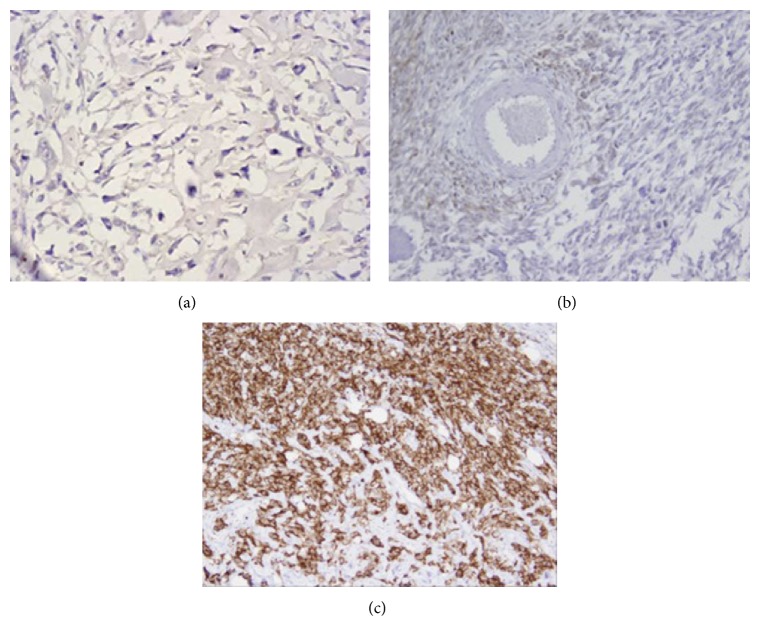
Immunohistochemical staining of B7-H4 in OS tissues and normal bone tissues. (a) Negative expression in normal bone tissues. (b) Weak positive staining of B7-H4 protein in OS tissues. (c) Strong positive staining of B7-H4 protein in OS tissues (×400 magnification).

We first analyzed B7-H4 protein expression in 104 OS specimens and corresponding normal bone tissues by immunohistochemistry. In normal bone tissues, B7-H4 staining was negative or weak. In OS tissues, positive B7-H4 staining was predominantly observed on the membrane and in cytoplasm of the tumor cells (Figure 1). Statistically, B7-H4 was high expressed in 70.19% (73/104) of OS tissues, which was significantly higher than the 14.4% (15/104) in normal bone tissues (*P* < 0.001) (Table 1).

### 3.2. Circulating sB7-H4 in Serum of Patients with OS

**Figure 2:**
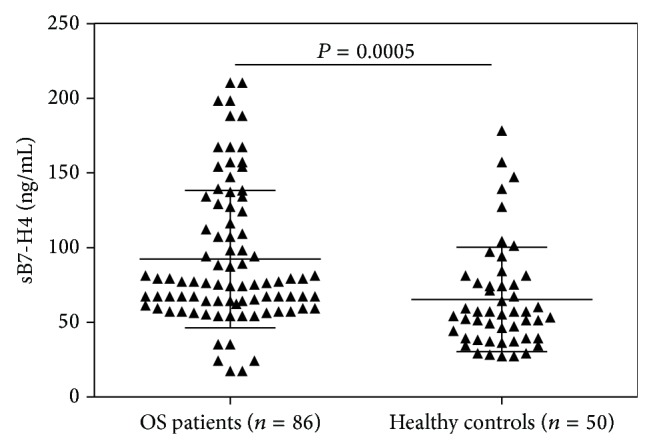
The levels of circulating sB7-H4 in the serum of OS patients and healthy controls were measured by ELISA. Mean concentration of sB7-H4 in patients with OS was significantly higher than that in healthy volunteers (92.28 ± 4.97 ng/mL versus 65.37 ± 5.22 ng/mL, *P* = 0.005).

Sandwich ELISA was used to examine the levels of sB7-H4 in serum of 86 patients with OS and 50 healthy controls. As shown in Figure 2, the mean level of sB7-H4 in serum samples from OS patients was 92.28 ± 4.97 ng/mL, which was significantly higher than that from healthy controls (65.37 ± 5.22 ng/mL) (*P* = 0.005).

### 3.3. Correlation between B7-H4 Expression and Clinicopathological Features

The relationship between B7-H4 protein expression (immunohistochemical staining) and clinicopathologic features was summarized in Table 2. There was no significant correlation of B7-H4 expression to age (*P* = 0.834), gender (*P* = 0.549), tumor site (*P* = 0.797), and differentiation status (*P* = 0.317). However, high expression B7-H4 was significantly correlated with advanced tumor stage (I + II versus III, *P* < 0.001) and distant metastasis (*P* = 0.034), suggesting that B7-H4 was critical in tumor progression and metastasis in OS.

The association of serum sB7-H4 levels with clinicopathological features of the patients with OS was also evaluated. As shown in Table 3, the mean level of sB7-H4 was significantly higher in patients with advanced tumor stage than in patients with early tumor stage (*P* = 0.017). Patients with distant metastasis had higher sB7-H4 levels when compared with those without distant metastasis (*P* = 0.021). However, there was no statistically significant correlation between sB7-H4 level and age (*P* = 0.404), gender (*P* = 0.517), tumor site (*P* = 0.674), and differentiation status (*P* = 0.127), respectively, suggesting that sB7-H4 levels may be closely associated with the development and progression of OS.

### 3.4. Correlation between B7-H4 Expression and Prognosis

**Figure 3:**
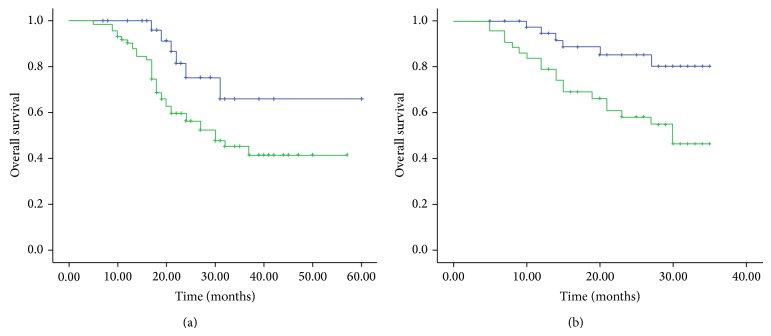
(a) Overall survival of 104 OS patients in relation to B7-H4 protein expression. (b) Overall survival of 86 OS patients in relation to sB7-H4 levels. Overall survival time was calculated using the Kaplan-Meier method and analyzed using the log-rank test.

The 5-year overall survival rates were 82.6% and 50.7% among patients with B7-H4 low and high expression (immunohistochemical staining), respectively (Figure 3(a)). Five-year overall survival rates were significantly lower in B7-H4 high than B7-H4 low patients (*P* = 0.028). In addition, as shown in Figure 3(b), the OS patients with high serum sB7-H4 levels had significantly shorter mean survival times (25.12 months), compared with OS patients with low sB7-H4 levels after diagnosis (31.57 months, *P* = 0.005).

Univariate analysis showed that tumor stage, distant metastasis, B7-H4 expression, and serum sB7-H4 levels were significantly related to overall survival (*P* < 0.001, *P* = 0.002, *P* < 0.001, and *P* = 0.001, resp., Table 4). Multivariate analysis showed that tumor stage, B7-H4 expression, and serum sB7-H4 levels were independent prognostic factors (*P* < 0.001, *P* = 0.004, and *P* = 0.041, resp., Table 4).

## 4. Discussion

B7-H4, a new member of the inhibitory B7 family, is regarded as a negative regulator of the T cell-mediated immune response. Previous studies have demonstrated that B7-H4 is highly expressed in many different types of human cancers and mostly associated with poor clinical outcomes [17]. However, the precise physiological function of B7-H4 and, especially, its role in the development and progression of human OS remain unidentified. One study reported that B7-H3, another member of B7 family molecules, was overexpressed in patients with OS and associated with tumor aggressiveness and metastasis [22], suggesting that costimulatory molecules play an important role in OS progression.

In the current study, we for the first time identified elevated expression levels of B7-H4 in both OS tissues and serum samples. B7-H4 was high expressed in 70.19% of OS tissues, significantly higher than the 14.4% in normal bone tissues (*P* < 0.001), while the mean level of sB7-H4 in serum samples from OS patients was significantly higher than that from healthy controls (*P* = 0.005). Our results were consistent with previous reports that B7-H4 was upregulated in numerous human malignancies [23] and blood samples from patients with ovarian cancer, gastric cancer, and renal cell carcinoma [18, 20, 21]. Therefore, B7-H4 and sB7-H4 might serve as a potential biomarker of various malignancies.

It is well documented that tumor size and tumor stage, as well as response to neoadjuvant chemotherapy, are important clinical characteristics in OS [24]. Several groups have reported that B7-H4 overexpression was frequently associated with many clinicopathological features, including tumor size, lymph node metastasis, depth of tumor invasion, and TNM stage [12, 14, 16], which also further gives prominence to the importance of B7-H4 in the development and progression of many tumors. In addition, Shi et al. reported that high sB7-H4 level was significantly correlated with tumor size, lymph node metastasis, and TNM stage in patients with gastric cancer [20]. To explore the possible roles of B7-H4 in OS, we also investigated the relationships between B7-H4 expression and different clinicopathological features. We found that both B7-H4 expression and sB7-H4 level are significantly correlated with advanced tumor stage and distant metastasis but not related to other characteristics, such as gender, age, tumor site, and differentiation status, indicating that B7-H4 may be a valuable biomarker for predicting tumor progression in patients with OS.

Because of the poor prognosis of patients with distant metastasis from OS, it is important to identify patients with OS who are at greater risk of developing distant metastasis. We found that patients with high B7-H4 expression in their primary OS have a higher risk of developing distant metastasis. Therefore, high B7-H4 expression in OS patients may indicate developing distant metastases and calls for more aggressive treatment and close surveillance. Moreover, higher sB7-H4 in blood samples from OS patients also correlated with distant metastases, indicating that serum B7-H4 may be a potential diagnostic marker for distant metastases of OS patients.

In our study, we further investigated the correlation between B7-H4 and prognosis of the patients with OS. Our findings revealed that B7-H4 in both tissues and serum samples was closely associated with overall survival of OS, and the survival time of the patients with elevated B7-H4 level was significantly lower than those with reduced B7-H4 level. Moreover, the multivariate analysis confirmed that B7-H4 in tissues and serum samples was an independent factor for affecting the survival time of OS patients. These results indicated that B7-H4 may be a novel prognosis predictor and therapeutic target of the patients with OS.

In summary, elevated B7-H4 level in tissues and serum samples may play critical roles in the development and progression of OS; detection of B7-H4 might serve as a clinical predictor in the diagnosis or prediction of clinical outcomes for the patients with OS. Further studies are needed to investigate the mechanism underlying the inhibitory function of B7-H4 and to further explore whether B7-H4 could be a useful target for the treatment of this disease.

## Supplementary Material

Patients, Specimens, and Follow-Up
Blood specimens were collected from 86 OS patients and 50 healthy controls at the Tianjin Hospital between 2010 and 2013. Patients who had undergone any form of preoperative chemotherapy and/or radiation therapy were excluded. The healthy controls were recruited from people who came for general health examinations. Selected characteristics of the cases and controls are presented in Table S1.

## Figures and Tables

**Table 1:** Expressions of B7-H4 in OS tissues and paired normal bone tissues.

Groups	Cases	Expression of B7-H4		*χ* ^2^	*P* value
		Low	High		
Tumor tissues	104	31	73	66.261	0.000
Normal tissues	104	89	15		

**Table 2:** Association between B7-H4 expression and clinicopathological features of 104 OS patients.

Characteristics	Cases	B7-H4 expression		*P* value
	(104)	Low (31)	High (73)	
Age (years)				
≤20	60	18	44	
>20	44	13	29	0.834
Gender				
Male	54	15	40	
Female	50	16	33	0.549
Tumor site				
Femur/tibia	67	20	49	
Others	37	11	24	0.797
Tumor stage				
I + II	62	24	29	
III	42	7	44	**0.000**
Distant metastasis				
Yes	41	13	47	
No	63	18	26	**0.034**
Differentiation status				
High	76	25	52	
Low	28	6	21	0.317

**Table 3:** Correlation between sB7-H4 levels and clinicopathological features of 86 OS patients.

Characteristics	Cases	Mean (range)	*P* value
	(86)	(ng/mL)	
Age (years)			
≤20	54	79.27 (35.7–157.6)	0.404
>20	32	87.29 (42.6–167.1)	
Gender			
Male	49	84.66 (24.3–188.2)	0.517
Female	37	97.34 (17.5–162.7)	
Tumor site			
Femur/tibia	62	97.24 (35.7–147.2)	0.674
Others	24	89.21 (44.3–154.3)	
Tumor stage			
I + II	64	79.2 (47.5–157.2)	**0.017**
III	22	124.1 (54.7–210.3)	
Distant metastasis			
Yes	25	128.2 (57.2–198.4)	**0.021**
No	61	81.27 (35.8–166.7)	
Differentiation status			
High	60	101.26 (34.4–164.2)	0.127
Low	26	81.17 (17.7–142.8)	

**Table 4:** Univariate and multivariate analyses of overall survival in OS patients.

Factors		Univariate			Multivariate	
	Hazard ratio	95% CI	*P*	Hazard ratio	95% CI	*P*
Age, years (≤20/>20)	1.017	(0.954–1.057)	0.479	1.177	(0.671–1.571)	0.759
Gender (male/female)	1.134	(0.718–2.271)	0.379	1.537	(0.519–1.871)	0.442
Tumor stage (I, II/III)	6.771	(2.177–13.079)	<0.001	6.147	(2.274–11.34)	<0.001
Distant metastasis (yes/no)	2.977	(1.374–5.127)	0.002	1.871	(0.757–2.622)	0.179
Differentiation status (high/low)	1.297	(0.674–2.774)	0.057	1.087	(0.633–1.997)	0.217
B7-H4 expression (high/low)	5.257	(2.224–9.274)	<0.001	3.147	(1.32–7.377)	0.004
sB7-H4 levels (high/low)^*^	4.371	(2.971–8.374)	0.001	2.022	(1.547–4.607)	0.041

^*^92.28 ng/mL as the cutoff value to divide OS patients into groups with high (*n* = 36) and low (*n* = 50) sB7-H4 levels.
