# Supplementary material for: B7-H4 Expression Is Associated with Tumor Progression and Prognosis in Patients with Osteosarcoma
Source: Biomed Res Int. 2015 Apr 14;2015:156432. doi: 10.1155/2015/156432 (PMC4411454; doi:10.1155/2015/156432)
Supplement: Supplementary file 1 — Patients, Specimens, and Follow-Up Blood specimens were collected from 86 OS patients and 50 healthy controls at the Tianjin Hospital between 2010 and 2013. Patients who had undergone any form of preoperative chemotherapy and/or radiation therapy were excluded. The healthy controls were recruited from people who came for general health examinations. Selected characteristics of the cases and controls are presented in Table S1. [file 156432.f1.pdf]

Table S1. General characteristics of the OS patients and healthy controls.

Characteristics	Osteosarcoma (n=86) (%)	Control (n=50) (%)
**Gender**		
Male	49 (56.9)	32 (64)
Female	37 (43.1)	18 (36)
**Age**		
≤20	54 (62.8)	31(62)
>20	32 (37.2)	19 (28)
**Tumor site**		
Femur	40 (46.5)	
Tibia	22 (25.6)	
Others*	24 (27.9)	
**Tumor stage**		
I	25 (29.1)	
II	39 (45.3)	
III	22 (25.6)	
**Distant metastasis**		
Yes	25 (29.1)	
No	61 (70.9)	
**Differentiation status**		
High	60 (69.8)	
Low	26 (30.2)	

*Others include Humerus (10 patients), pelvic (6 patients), radius (5 patients) and ulna (3 patient).
